# The role of gut microbiota in insulin resistance: recent progress

**DOI:** 10.3389/fmicb.2025.1633029

**Published:** 2025-07-25

**Authors:** Hongfang Ji, Shenglin Su, Min Chen, Shuhong Liu, Shu Liu, Jianjin Guo

**Affiliations:** ^1^Third Hospital of Shanxi Medical University, Shanxi Bethune Hospital, Shanxi Academy of Medical Sciences, Tongji Shanxi Hospital, Taiyuan, China; ^2^Department of Critical Care Medicine, General Hospital of Ningxia Medical University, Yingchuan, China

**Keywords:** insulin resistance, gut microbiota metabolism, carbohydrate fermentation, host-microbiota crosstalk, short-chain fatty acids

## Abstract

Insulin resistance (IR) is the most critical pathophysiological basis of type 2 diabetes mellitus (T2DM). The mechanisms underlying IR are extremely complex, with variations observed among different patients and different tissues. Current research has gradually revealed characteristics of the gut microbiota and its role in metabolizing major nutrients during IR. The interaction between microbial communities and host metabolism has become a hotspot in modern medical research. This review aims to provide a comprehensive understanding of microbial metabolism related to IR by elucidating the effects of microbiota-carbohydrate metabolism and microbiota-host interactions on IR. Such insights may contribute to improving IR and offer novel perspectives for its treatment.

## 1 Introduction

According to the latest Global Diabetes Map released by the International Diabetes Federation (IDF), up to 589 million adults aged 20–79 will have diabetes in 2024. More worryingly, this number is expected to rise to 853 million by 2050, representing approximately one-eighth of the world’s population (International Diabetes Federation, 2025). This underscores the urgency of developing novel therapeutic approaches. Insulin resistance (IR), as a key component of type 2 diabetes (T2D), is regulated by multiple factors, including genetics, environment, and lifestyle. The intestinal microbiota, referred to as the “second genome” and a critical “metabolic organ,” has garnered increasing attention for its roles in energy metabolism regulation and immunomodulation. Abundant evidence demonstrates that dysbiosis and functional impairments in the gut flora, along with alterations in their metabolite profiles, are closely associated with the onset and progression of IR. Elucidating the role of gut microbes in IR, particularly their carbohydrate metabolic pathways and their intricate interactions with the host, not only enhances our understanding of IR’s pathophysiology but also offers potential targets for developing novel IR intervention strategies based on the modulation of intestinal microbiota. In this review, we focus on two core areas: the gut microbial–carbohydrate metabolism axis and microbial–host interactions. We systematically review recent advances in their association with IR, discuss the clinical application prospects and challenges, and aim to provide innovative insights into the prevention and treatment of type 2 diabetes mellitus (T2DM) and related metabolic diseases.

Literature was retrieved from PubMed, Web of Science, and Scopus on 14 June 2024, using the following keywords: “insulin resistance,” “gut microbiota metabolism,” “short-chain fatty acids,” and “carbohydrate fermentation.” Only peer-reviewed articles published in English between 2000 and 2025 were considered. The inclusion criteria included studies focusing on IR and microbial metabolism, experimental studies or systematic reviews, and articles published in English. Exclusion criteria were as follows: non-English articles, non-peer-reviewed articles, and conference abstracts without full text. The selection process involved a two-step screening procedure: first, titles and abstracts were reviewed to exclude irrelevant studies; second, full-text articles were assessed for eligibility.

## 2 Relevant sections

### 2.1 Gut microbiota and human metabolism

The gut microbiota, a complex ecosystem of trillions of microorganisms, serves as a critical regulator of human health and disease. It comprises bacteria, viruses, fungi, and protozoa, with the majority colonizing the gastrointestinal tract. The cecum and proximal colon harbor the highest microbial biomass and diversity, followed by the small intestine, while the remaining populations occupy distinct ecological niches such as the skin and oral cavity. The gut microbiome (collective genetic material of the microbiota) contains nearly 1,000 times more genes than the human genome—over 22 million microbial genes compared to 23,000 human genes—endowing this community with remarkable metabolic versatility. The core microbiome, a conserved set of functional genes across individuals, underpins essential biological processes and plays a pivotal role in maintaining the dynamic equilibrium of the gut ecosystem (Wu et al., 2024).

#### 2.1.1 Gut microbiota–carbohydrate metabolism

The diversity of gut microbiota confers a unique advantage in carbohydrate degradation, with the rich and diverse microbial community functioning through cooperative-competitive dynamics. Humans lack most enzymes required for carbohydrate breakdown, while gut-dwelling microorganisms depend on carbon sources for energy. Consequently, these microbes produce enzymes to metabolize carbohydrates, fulfilling their own energy demands and supporting the host in acquiring essential energy—contributing to nearly 10% of the body’s total energy intake (Karlsson et al., 2013). Enzymatic reactions are pivotal drivers of carbohydrate degradation within the gut microbiota. Gut microbes are capable of encoding over 100 different carbohydrate-active enzymes that break down a variety of complex polysaccharides, such as cellulose and hemicellulose (Zhang H. et al., 2020). The evolution of specific CAZymes (carbohydrate-active enzymes) provides a competitive advantage to certain bacteria, leading human gut bacteria to dedicate a significant portion of their genome to expressing these enzymes. This allows them to facilitate their energy supply by breaking down carbohydrates through specific enzymatic reactions (Wardman et al., 2022; Li et al., 2023; Nie et al., 2023; Takeuchi et al., 2023). Approximately 30%–40% of dietary fiber is not digested or absorbed in the small intestine but is instead fermented by gut microbiota in the large intestine. When faced with complex carbohydrates (e.g., polysaccharides and cellulose) that evade catabolism by host enzymes in the small intestine and pass to the distal gut, microorganisms possessing specific enzymes metabolize these complex carbohydrates through a series of complex enzymatic reactions. For instance, the phylum Bacteroidetes dedicates a significant portion of its genome to glycoside hydrolases and polysaccharide-cleaving enzymes, utilizing thousands of enzyme combinatorial forms to dominate the gut microbiome (Lapébie et al., 2019; Ye et al., 2021). In the degradation of simple carbohydrates, such as glucose and fructose, gut microbes exhibit the same metabolic flexibility. These mono- and disaccharides are rapidly absorbed in the gut, and the unabsorbed portion serves as an energy source for gut microbes. Lactobacillus and Bifidobacterium are capable of fermenting these simple sugars to produce lactic acid and other short-chain fatty acids (SCFAs). These products not only provide energy for the gut microbes themselves but also play a key role in maintaining the pH balance of the gut and inhibiting the growth of harmful flora (Cani et al., 2019). An in-depth study of enzymes encoded by intestinal flora can help us better understand the symbiotic relationship between flora and the human body. However, there remains a significant quantitative discrepancy between the enzymes of human intestinal flora origin mined so far and the discovered human intestinal flora genomic data, which highlights the research value and mining potential of this area.

Short-chain fatty acids, particularly butyrate and propionate, are pivotal microbial metabolites orchestrating systemic energy balance and glucose homeostasis. The mechanisms by which SCFAs influence IR primarily center on enhancing insulin sensitivity, modulating intestinal barrier function, exerting anti-inflammatory effects, and regulating energy metabolism. Animal studies have demonstrated that SCFAs promote the secretion of glucagon-like peptide-1 (GLP-1) from intestinal L-cells, which activates the GLP-1 receptor in the vagus nerve, thereby inducing satiety signaling in the hypothalamus. GLP-1 enhances glucose-stimulated insulin secretion (GSIS), leading to a reduction in circulating glucose levels, while simultaneously inhibiting glucagon secretion and decreasing endogenous glucose production. These effects contribute to reduced food intake and delayed gastric emptying (Yadav et al., 2013). Butyrate may improve insulin sensitivity by promoting insulin secretion through the activation of G protein-coupled receptors (GPR43, GPR41, among others) and inhibiting hepatic gluconeogenesis (Cong et al., 2022; Moran et al., 2016). Butyrate supports the integrity of the intestinal barrier by stimulating the proliferation of epithelial cells and upregulating the expression of tight junction proteins, such as occludin and zona occludens-1 (Yan et al., 2023). Furthermore, it enhances barrier function by facilitating the interaction between the transcription factor SP1 and the Claudin-1 promoter, thereby increasing Claudin-1 transcription (Wang et al., 2012). Animal experiments have demonstrated that SCFAs influence human islet cell viability in a concentration-dependent manner. Specifically, acetic acid and butyric acid at physiological concentrations (1 and 2 mmol/L) inhibit β-cell apoptosis and prevent a reduction in the oxygen consumption rate by supporting mitochondrial respiratory function (Hong et al., 2016; Layden et al., 2013). Additionally, SCFAs modulate energy metabolism by activating AMP-activated protein kinase (AMPK) and other energy sensors, thereby promoting fat oxidation and glucose utilization and improving IR (Kimura et al., 2013). Furthermore, SCFAs reduce systemic inflammation and alleviate IR by suppressing the expression of pro-inflammatory cytokines [e.g., interleukin-6, IL-6, and tumor necrosis factor-α (TNF-α) and reducing the translocation of endotoxins [e.g., lipopolysaccharides (LPS)] from the intestine into the bloodstream (Zaky et al., 2021). The aforementioned mechanisms are primarily derived from studies conducted in preclinical animal models and *in vitro* experimental settings. The findings described above were obtained under specific experimental conditions, and their applicability to humans remains to be further validated. Moreover, these studies establish a correlation between SCFAs and IR but do not directly demonstrate a causal relationship.

This spatial-functional specialization highlights the microbiota’s role as a metabolic “second liver,” transforming dietary residuals into bioactive mediators with pleiotropic host benefits. A multi-omics analysis of 952 normoglycemic individuals—integrating host genotypes, gut metagenomic profiles, fecal SCFA levels, and 17 metabolic/anthropometric parameters—revealed two key associations: Elevated butyrate levels were positively associated with enhanced insulin response (*P* = 9.8 × 10^–5^). Increased fecal propionate (indicative of dysregulated microbial production or impaired host absorption) was linked to higher risk of T2D (*P* = 0.004) (Sanna et al., 2019). Consistent with these findings, recent studies report elevated propionate levels in fecal carbohydrate metabolites of insulin-resistant patients (Takeuchi et al., 2023). While numerous studies suggest that gut microbial-derived SCFAs may confer anti-obesity and antidiabetic benefits to the host (Chambers et al., 2015; Zhao et al., 2018). Paradoxical evidence from both *in vitro* and *in vivo* models indicates that dysregulated SCFA accumulation might exacerbate metabolic dysfunction (Peng et al., 2007; Schwiertz et al., 2010; Zhang et al., 2025). This duality underscores the need for multidimensional analytical frameworks to: decipher context-dependent effects: elucidate how microbial community structure, host genetics, and dietary patterns jointly modulate SCFA bioactivity. Establish causal mechanisms: integrate multi-omics datasets to disentangle SCFA-mediated host-microbe metabolic crosstalk. Such approaches will clarify the therapeutic potential of SCFAs while addressing current controversies in gut microbiota research.

#### 2.1.2 Gut microbiota–host metabolic interactions

The interaction between microbial communities and host metabolism has become a prominent focus in modern medical research. Over the past decade, significant advances have been made in understanding the composition and functional dynamics of complex microbial communities inhabiting the gastrointestinal tract, as well as those colonizing the skin and oral cavities of humans and animals (Hou et al., 2022). Scientific consensus now recognizes that microorganisms exist in symbiotic equilibrium with their hosts under healthy conditions, exerting profound influences through three primary mechanisms: regulation of nutrient metabolism, protection against pathogenic invasion, and modulation of immune cell signaling to maintain physiological homeostasis and enhance immune competence (de Vos et al., 2022).

While investigations into the determinants of microbiome diversity remain ongoing, integrated methodologies encompassing metabolomics, genomics, and fecalomics have catalyzed a continuum of scientific advancements and pivotal discoveries in recent years:

This environmental priming of commensal communities during prime developmental windows highlights the actionable potential of microbiome-targeted preventive strategies. Host-microbiota protein interactome mapping reveals unprecedented transkingdom connectivity. Yale investigators pioneered BASEHIT (Biotin-Assisted Screening of Ectoprotein-Hosted Interactions via Transkingdom coculture), an integrative platform combining: systematic co-culture of biotinylated bacterial isolates with human ectoprotein-expressing yeast libraries; affinity-based isolation of microbe-host complexes; high-throughput sequencing for interaction partner resolution. Application of this multi-omics approach decoded 1,724,589 candidate binding events (519 commensal strains × 3,324 ectoproteins), with 68.3% representing novel interactions beyond existing databases. This landscape illuminates the staggering scale of evolved molecular crosstalk at the host-microbe interface. Notably, the interaction network reveals a conserved logic of molecular recognition: homozygous microbial strains exhibit convergent binding to evolutionarily conserved exoprotein hubs, while tissue-specific isolates selectively engage microenvironment-specific exoproteins such as gut lumen-dominant digestive enzymes or airway surface liquid-enriched protease inhibitors. These studies elucidate previously uncharted host-microbiota interactions at the molecular level (Sonnert et al., 2024).

**FIGURE 1:**
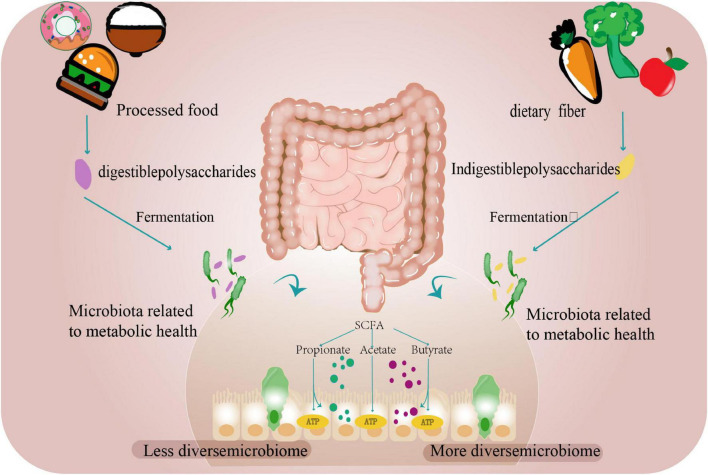
The gut microbiome and diet. Dietary carbohydrates and proteins undergo microbial fermentation in the colon. Digestible components are processed by host enzymes, while resistant fibers and polysaccharides serve as substrates for gut microbiota to produce SCFAs, including acetate, propionate, and butyrate. These metabolites function as energy sources while supporting microbial community stability. Ultra-processed foods exhibit reduced fiber content due to industrial processing, correlating with decreased microbial diversity. In contrast, adequate fiber intake promotes microbial richness and balanced bacterial populations.

Empirical evidence from a European cohort study substantiates the ecological paradigm: early-life exposure to agrarian microbial biodiversity profoundly modulated intestinal ecosystem assembly, demonstrating accelerated microbiota maturation trajectories during the critical first postnatal year (Depner et al., 2020). Not only the external environment, but also changes in the host’s internal digestive environment can impact the intestinal flora. Recent multinational studies demonstrate that plant-derived fibers play a critical role in maintaining microbial diversity, as distinct dietary patterns shape specific gut microbiota profiles. Longitudinal investigations reveal that individuals adhering to plant-based diets exhibit elevated levels of fiber-fermenting and SCFA-producing bacteria (e.g., *Faecalibacterium* and *Roseburia*) during vegan interventions. These microbial alterations correlate with reduced systemic inflammation (quantified by decreased pro-inflammatory cytokines) and suppression of oncogenic signaling pathways through epigenetic mechanisms. Current evidence supports dietary fiber augmentation as a viable strategy for modulating gut microbial ecology and enhancing intestinal homeostasis (Yin et al., 2025; Fackelmann et al., 2025; Nshanian et al., 2025; Li et al., 2024; Figure 1).

Emerging research has increasingly focused on elucidating microbial-host interaction mechanisms through genomic approaches. Studies indicate that adults exhibiting low gut microbial gene richness demonstrate elevated risks of IR, pro-inflammatory lipid profiles, and adiposity compared to those with high gene richness (Liu et al., 2017). The ABO locus ranks among the most frequently identified genetic determinants in gut microbiota studies, with robust associations observed between host blood types and metabolic health. This linkage appears particularly pronounced in individuals expressing mucosal A antigens, where host genetic influences on gut microbial communities likely operate through environment-host factor interactions (Qin et al., 2022; Zhang et al., 2024; Lopera-Maya et al., 2022). Bacterial structural variants (SVs) drive functional diversification by enhancing genomic plasticity, a critical mechanism facilitating microbial adaptation to gut environments (Aras et al., 2003). Yet the role of host genetic factors in shaping gut ecosystem dynamics that select for such adaptive microbiome evolution remains mechanistically underexplored. A population-scale association analysis of human genetic variation and gut microbial structural variation across 9,015 individuals identified a robust correlation (Minimum *P*-value = 4.19 × 10^–223^) between blood group A phenotype (encoded by the ABO gene) and a conserved genomic locus harboring N-acetylgalactosamine (GalNAc) catabolic operons in *Faecalibacterium prausnitzii* (phylum Firmicutes). *In vitro* functional validation demonstrated that GalNAc utilization as a sole carbon source was strictly dependent on intact GalNAc catabolic operons in *F. prausnitzii* (Firmicutes) (Zhernakova et al., 2024). Mechanistically, ABO blood group determinants mediated bacterial genomic restructuring through GalNAc-mediated selection pressure, providing evolutionary evidence for host genetic regulation of gut microbial ecology. Notably, empirical evidence supporting host genetic modulation of microbial functional hierarchies remains scarce. Future investigations should prioritize bridging the methodological gap between conventional species abundance metrics and functional validation frameworks, particularly through integrating metagenomic signatures with mechanistic insights from transcriptomic/proteomic profiling. Analysis of microbial genome studies reveals that host genetics governs specific components of the microbiome, whereas environmental perturbations prompt structural genomic adaptations through microbial-driven gene reorganization. This functional specificity constitutes a key determinant of inter-individual variability, emerging from the interplay of host genetics and environmental factors. Current evidence cannot definitively establish the universal host-beneficial nature of such genomic evolution across developmental stages. However, empirical observations identify select SVs with measurable health impacts, presenting mechanistic complexities requiring systematic resolution through multi-omics investigations.

The persistence of complex microbial communities within mammalian hosts occurs despite continuous immune surveillance, with evolutionary maintenance of gut homeostasis rather than pathogenic eradication. Substantial evidence reveals phylogenetically conserved host-microbiota crosstalk across species, particularly through gene-microbiome interactions that exert quantifiable immunomodulatory effects (Zhang et al., 2024). Notably, these reciprocal regulatory networks demonstrate context-dependent activation patterns, though the molecular mechanisms governing this dynamic equilibrium remain incompletely characterized (Rollenske et al., 2021).

The gut-brain, gut-liver, and gut-lung axes have been increasingly investigated in recent years. Advancements in microfluidic systems and cell biology techniques have facilitated the development of *in vitro* gut microbiota models, enabling systematic exploration of their interactions with distal organs through multidirectional axes. This progress provides a framework for elucidating microbial functional diversity and accelerating clinical translation (Hall et al., 2024; Lloyd, 2024; Barone et al., 2022). This multi-scale approach will ultimately construct a dynamic microbial ecosystem atlas, providing a theoretical framework for precise interventions in multi-organ diseases.

### 2.2 IR and gut microbiota regulation of host metabolism

#### 2.2.1 Defining IR: from molecular mechanisms to translational advances

Insulin resistance is a pathophysiological state characterized by impaired sensitivity and responsiveness to endogenous or exogenous insulin in insulin-target tissues (skeletal muscle, liver, and adipose tissue) under homeostatic conditions, leading to suboptimal efficiency of insulin-mediated glucose uptake and utilization. Evidence indicates that IR exhibits a stronger association with diabetes risk compared to β-cell dysfunction in Chinese and other East Asian populations, with this association being particularly pronounced among individuals with obesity. Epidemiological analyses reveal that the Chinese population demonstrates heightened susceptibility to diabetes mellitus at lower body mass index (BMI) thresholds compared to Western populations (Wang et al., 2020). IR is not only closely associated with other components of the metabolic syndrome—including obesity and non-alcoholic fatty liver disease—but also increases the risk of cardiovascular disease (Muzurović et al., 2021). Given the complex mechanisms underlying IR, recent advances in related studies contribute to a more comprehensive understanding of its pathophysiology.

Researchers at Fudan University first analyzed BACH1 expression patterns through GEO database mining. Single-cell RNA sequencing of liver cells identified significant upregulation of BACH1 transcripts in mice fed a high-sucrose diet compared to controls (standard chow diet). Subsequent orthogonal validation showed consistent BACH1 elevation in: (i) hepatic tissue from diet-induced obese mice (high-sucrose-and-high-fat diet); (ii) leptin receptor-deficient (db/db) diabetic mice; and (iii) primary hepatocytes treated with oleic acid (OA, 1 mM) for 24 h. These multi-model observations suggest BACH1 may mediate metabolic stress responses during IR development (Jin et al., 2023).

Researchers at the Institut de Recherche Clinique de Montréal (IRCM) conducted experiments investigating growth arrest-specific secretory protein 6 (GAS6). Their data revealed that GAS6-deficient mice exhibited complete prevention of high-fat/high-sugar diet-induced IR. Conversely, pharmacological elevation of circulating GAS6 levels directly triggered IR. This study provides first experimental evidence of a causal relationship between the GAS6 signaling axis and IR pathogenesis. Emerging research indicates that GAS6 and its cognate receptor AXL (tyrosine-protein kinase receptor) may represent therapeutic targets for IR. Mechanistically paralleling insulin’s action, GAS6 signals through AXL receptor binding. This interaction implies that AXL may form complexes with insulin receptors and that GAS6 binding to muscle cell receptors induces insulin response reprogramming rather than maintaining normal sensitivity (Schott et al., 2024). A recent study has proposed an intriguing idea through mathematical modeling and analysis of the insulin signaling pathway: the microscopic basis of IR is the cellular threshold response to insulin. That is, insulin must surpass this threshold to become effective, and this threshold can serve as a microscopic definition of IR. To prevent hyperglycemia and hypoglycemia, muscle glucose uptake exhibits a bistable response. This response involves two thresholds: an opening threshold and a closing threshold. The opening threshold is significantly higher than the fasting insulin level, minimizing the risk of hypoglycemia. When a large amount of food is consumed, the rising insulin level eventually exceeds the opening threshold, prompting muscles to take up glucose at a maximum rate. This uptake begins to reverse when insulin levels peak, drop below the opening threshold, and continue to fall. Only when insulin levels fall below the closing threshold does muscle glucose uptake cease. The threshold effect causes muscle tissue to delay glucose uptake, which might seem to increase the risk of hyperglycemia. However, this delay actually maximizes glucose uptake efficiency through a compensatory mechanism. During the waiting period, the accumulated peak glucose concentration fully activates the transporter system (Akhtar et al., 2024). A study tested this bistability by combining cellular experiments with a mathematical modeling hypothesis, and the results suggest that the body’s ability to avoid both hypoglycemia and hyperglycemia is mediated by this bistable response (Akhtar et al., 2022). The opening threshold is a promising biomarker for metabolic complications due to its strong quantitative link with body composition. However, these innovative theoretical frameworks still lack substantial experimental validation.

In summary, evidence indicates that distinct dietary patterns and activation of biological targets induce specific signaling pathway activations, which drive IR and disrupt glucose, protein, and adipose tissue metabolism, ultimately impairing whole-body energy homeostasis. Current evidence remains confined to laboratory studies and awaits verification through large-scale clinical trials.

#### 2.2.2 Gut microbial substrate metabolism and IR

**FIGURE 2:**
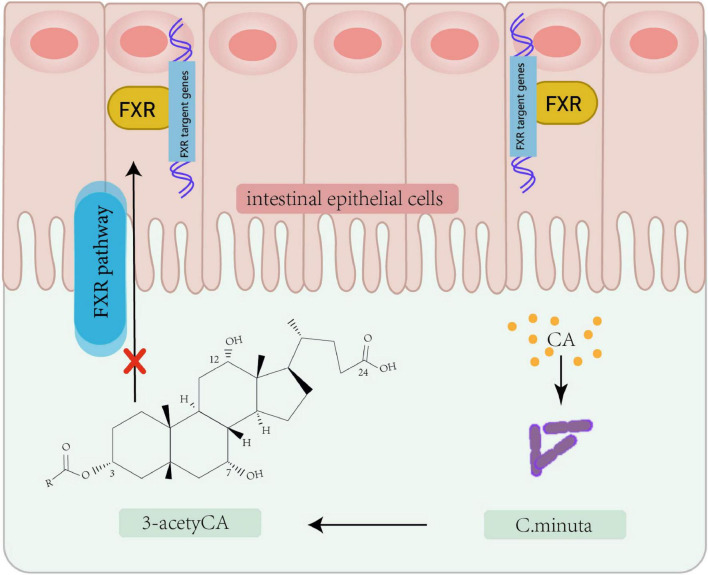
Bacterial modification of cholic acid leading to FXR pathway inhibition. *Christensenella minuta*, modifies cholic acid (CA) to 3-acetylCA. This 3-acetylCA then inhibits the farnesoid X receptor (FXR) pathway in intestinal epithelial cells.

In recent years, microbial modulation has emerged as a focus of increasing research interest for addressing IR. As a central pathological feature of T2D and metabolic syndrome, IR development involves multifactorial mechanisms encompassing genetic predisposition, lifestyle factors, and gut microbiota composition and functionality. Microbial metabolomics studies have demonstrated that decreased abundance of *Akkermansia muciniphila* correlates with impaired insulin secretion, with significantly lower levels observed in new-onset diabetes patients and more severe secretory dysfunction in those lacking this bacterium. Multiple microbial metabolites demonstrate strong associations with the pathogenesis of IR. Imidazole propionate (ImP), a histidine-derived bacterial metabolite, induces IR (Fan and Pedersen, 2021). Mice fed a phenylalanine-rich diet, phenylalanine-producing aspartame, or engineered to overexpress human phenylalanine-tRNA synthetase (hFARS) develop IR and T2D symptoms (Zhou et al., 2022). Whether regulating the activity of these gut microbes and their metabolites to treat IR could become a biomarker for early diagnosis and therapy. Previous studies have revealed the role of metabolites including lipids, amino acids, and bile acids in regulating insulin sensitivity through metabolomic and lipidomic analyses of large datasets. Adipose tissue regulates insulin by secreting insulin-sensitizing factors (e.g., lipocalins) and storing lipids. Bäckdahl et al. (2021) revealed that human white adipose tissue contains three mature adipocyte subtypes, with only one demonstrating insulin sensitivity. Microbial-derived succinic acid enhances adipose thermogenesis via uncoupling protein 1 (UCP1) expression, while concurrently inducing macrophage-mediated pro-inflammatory responses through TLR4 activation, potentially triggering adipose inflammation and subsequent IR (Fan and Pedersen, 2021; Table 1). The gut commensal bacterium *Christensenella minuta* modulates host metabolism via producing a novel class of secondary bile acids featuring 3-O-acyl substitutions (Figure 2), which act as potent inhibitors of intestinal farnesoid X receptor (FXR) signaling. These 3-O-acyl-cholic acids are detectable at stable levels in normoglycemic individuals but exhibit significant depletion in T2D patients (Liu et al., 2024; Mohanty et al., 2024). Emerging evidence highlights a tightly regulated bidirectional crosstalk between bile acids and gut microbiota: bile acids shape microbial community structure and functional gene expression, whereas gut microbiota reciprocally fine-tune bile acid profiles through biotransformation processes, ecological network remodeling, and modulation of host cholesterol-derived metabolite signaling (Guzior et al., 2024; Jia et al., 2024; Sato et al., 2021; Wang et al., 2021). Animal studies have also found that bile acid derivatives such as porcine-derived deoxycholic acid (DCA) and hyodeoxycholic acid (HDCA), which exhibit significant depletion in T2DM cohorts (Jia et al., 2025). The metabolism of specific bile acid species plays crucial roles in maintaining intestinal homeostasis, providing critical insights into microbial metabolic contributions to host physiology.

**TABLE 1:** Products of gut microbial fermentation of carbohydrates.

Bacteria and genera	Metabolite	Representative substances	Physiological functions
*Bacteroides, Firmicutes, Akkermansia muciniphila*	SCFAs	Acetate, propionate, butyrate	Maintain intestinal barrier integrity, exert anti-inflammatory effects, regulate immune function, provide energy for intestinal epithelium, enhance insulin sensitivity (Chambers et al., 2015; Zhao et al., 2018)
*Clostridium, Bacteroides, Christensenella minuta*	Secondary bile acids	Deoxycholic acid, lithocholic acid	Regulate lipid metabolism, modulate host bile acid signaling pathway (Guzior et al., 2024; Jia et al., 2024; Sato et al., 2021; Wang et al., 2021)
		3-O-acylated secondary bile acids	Targetedly inhibit intestinal FXR, regulate the gut-liver signaling axis, alleviate glucose and lipid metabolic disorders (Liu et al., 2024)
*Lactic acid bacteria, Bifidobacterium, Clostridium*	Tryptophan metabolites	Indole, indole-3-propionic acid, 5-hydroxytryptophan	Modulate immune responses, maintain intestinal barrier integrity, facilitate neurotransmitter synthesis (e.g., serotonin)
*Bacteroides*	Histidine derivatives	Imidazole propionate	Inhibit insulin receptor signaling (Fan and Pedersen, 2021)
*Bacteroides, Escherichia coli*	Vitamin	Vitamin K, B vitamins (e.g., B12)	Mediate coagulation processes, regulate energy metabolism, support neurological function
*Escherichia coli, Lactobacillus*	Phenylalanine	Phenylalanine	positively correlated with HOMA-IR index (Zhou et al., 2022)

In addition, dietary modifications, probiotics, and prebiotics can promote the growth of beneficial microorganisms, inhibit harmful ones, and ameliorate metabolic disorders. Probiotic administration has been shown to affect clinical markers of T2D in animal models and randomized controlled trials (RCTs) (Qu et al., 2024; Wang et al., 2022; He et al., 2023). Patients receiving probiotic supplementation exhibited significantly lower glycated hemoglobin levels and improved indices of pancreatic β-cell function compared to those taking metformin alone (Chen et al., 2023). However, both animal and clinical studies consistently indicate that probiotic-induced alterations to the gut microbiota may not always benefit host metabolism. These alterations carry the potential for negative effects, primarily through the inhibition of other commensal microorganisms. As a novel prebiotic, phloretin (an apple-derived phytonutrient) has demonstrated ameliorative effects on IR in mouse models. High-throughput sequencing and fecal transplantation revealed this occurs via modulation of the “gut microbiota-barrier axis.” Despite phloretin’s low bioavailability, its combination with effector molecules (e.g., SCFAs) enhances therapeutic efficacy in mice fed a high-fat diet (Zhang X. et al., 2020). Furthermore, supplementing with additional microorganisms alongside prebiotics may prove significantly more effective than using probiotics alone.

Causal associations between the imbalance of microbial communities and IR have become a focal point in metabolic disorders research. Patients with obesity and T2D often exhibit reduced gut microbial diversity and an enrichment of specific bacterial species (Liu et al., 2017). Alterations in the composition and function of the gut microbial community, known as dysbiosis, are closely associated with the metabolic status of the host. In one study, researchers collected feces from human twin volunteers with significant differences in obesity for microbial community isolation and culture, followed by mouse fecal transplantation. The results showed that the microbial community of obese mice could lead to weight gain and decreased insulin sensitivity in lean mice (Ridaura et al., 2013). Similarly, another study collected human infant fecal samples for colonization in mice and found that mice colonized with fecal samples from infants aged 7–12 months had significantly higher insulin-expressing tissues and serum insulin levels. These findings suggest the presence of microorganisms that promote B-cell growth in humans between the ages of 7 and 12 months. It is likely that there is a specific developmental window in humans during which certain microorganisms promote B-cell proliferation and may regulate metabolic health throughout life (Hill et al., 2025). A Japanese study employing multi-omics integrative analysis revealed gut microbiome involvement in IR. Data indicated elevated fecal carbohydrates (e.g., fructose, galactose, mannose, and xylose) in insulin-resistant individuals and further demonstrated that insulin sensitivity-associated bacterial taxa ameliorate host IR phenotypes in murine models. To corroborate these findings, re-analysis of previously published fecal metabolomic data from the Twins UK cohort revealed that fecal monosaccharides—specifically glucose and arabinose—were positively correlated with both obesity and HOMA-IR (a validated metric for IR), both of which exhibited associations with IR (Takeuchi et al., 2023).

However, a number of human fecal microbiota transplantation (FMT) trials have revealed heterogeneous results and highlighted the limitations of current technical approaches to varying degrees. For instance, low fermentable cellulose combined with a single oral FMT capsule has been shown to improve insulin sensitivity in patients with severe obesity and metabolic syndrome (Mocanu et al., 2021). However, some studies have demonstrated significant variation in donor flora colonization rates in the recipients’ intestines, with differences as high as 40% between allogeneic and autologous transplantation (Kootte et al., 2017). This variation directly impacts therapeutic outcomes and explains why some clinical patients do not experience a beneficial effect following FMT. Furthermore, donor-recipient interactions, variations in microbiota composition among donors, and even temporal changes in the microbial composition of donors themselves can unpredictably influence the effectiveness of FMT (Sorbara and Pamer, 2022). Current technological tools are insufficient to overcome limitations related to the “expiration” of gut microbial communities; a single transplant may only sustain microbial changes for a few weeks. Additionally, there is a lack of standardized protocols for the frequency of repeat transplants.

### 2.3 Gut microbe-based clinical applications in IR: prospects and limitations

In summary, gut microbes can promote the catabolism of energy products, such as carbohydrates and fats, by producing specific enzymes or inducing their own gene mutations. These processes help maintain the balance of energy intake, their own homeostasis, and perform other roles. The study of gut microbes offers new insights and potential targets for the diagnosis and treatment of IR. Currently, the host microbiota involved in IR undergo dynamic changes in response to various factors, increasingly pointing toward personalized nutrition and probiotic-based therapies. Emerging technologies, ranging from dietary interventions to the use of probiotics and FMT, provide diverse options for treating IR. Given the currently observable changes, metabolites may serve as more reliable or accurate clinical endpoints for the prevention and treatment of IR compared to microbial taxa. This is because metabolites represent the cumulative output of microbial functions, sometimes even involving host-microbiota co-metabolism.

The composition and function of the gut microbiota exhibit significant inter-individual variability, posing substantial challenges in standardizing diagnostic and therapeutic strategies based on gut microbiota profiles. Current understanding of the effects of long-term probiotic interventions remains limited, as the functional characteristics of probiotic strains can lead to individual differences in their ability to colonize and metabolize within the gut. Consequently, existing studies predominantly focus on short-term interventions, while research on the long-term regulation of the gut microbiota faces difficulties in sustaining the effects of prolonged interventions. This is attributed to the limited colonization ability of probiotic strains in the gut and the potential influence of other metabolic regulatory pathways, whose long-term effects are uncertain. Moreover, the paucity of large-scale, high-quality data from long-term clinical trials underscores the lack of evidence supporting the sustained improvement of IR through long-term probiotic interventions. Presently, most evidence remains at the associative level, emphasizing the need for more robust studies. Specifically, well-designed longitudinal cohort studies and RCTs are essential to establish causality, particularly those that track the temporal association between changes in colony dynamics and metabolic indicators. Such research is crucial for advancing our understanding and developing effective interventions in the field of gut microbiota and metabolic health.

The available evidence indicates a strong correlation and partial causal association between gut microbiota and IR, though a strictly unidirectional causal relationship has not been conclusively established. The primary challenges include bidirectional interactions and entanglement, requiring further exploration of the mechanisms linking dysbiosis and IR. Additionally, population heterogeneity presents a significant challenge, as the contribution of gut microbiota to IR may vary considerably, influenced by genetic background (e.g., T2DM risk genes), dietary patterns, and other factors. Future research should employ Mendelian randomization studies and multi-omics longitudinal analyses to determine the direction of causality while controlling for confounding factors, rather than merely assuming that “flora causes IR.” Translating basic research into clinical application and conducting large-scale clinical trials to assess safety and efficacy face several challenges, including the need to improve the accuracy of microbiomics technology, clarify the mechanisms underlying individual differences in intervention effects, address the potential for adverse reactions and complex causal relationships, and ensure the safety of long-term interventions. Achieving precision therapy remains a goal that modern molecular biology has yet to fully attain. However, advancements in molecular biology techniques are increasingly being utilized in research, enabling the identification of microorganisms crucial for degrading specific carbohydrates. These techniques also reveal the genetic potential of the intestinal microbial community, providing genetic information on its microorganisms. This data facilitates the analysis of functional diversity in carbohydrate metabolism, thereby laying the groundwork for personalized treatment strategies.

## References

[B1] Akhtar J. Han Y. Han S. Lin W. Cao C. Ge R. (2022). Bistable insulin response: The win-win solution for glycemic control. *iScience* 25:105561. 10.1016/j.isci.2022.105561 36465102 PMC9708922

[B2] Akhtar J. Imran M. Wang G. (2024). Protocol for live-cell Förster resonance energy transfer imaging to reveal the bistable insulin response of single C2C12-derived myotubes. *STAR Protoc.* 5:103109. 10.1016/j.xpro.2024.103109 38829736 PMC11179099

[B3] Aras R. Kang J. Tschumi A. Harasaki Y. Blaser M. (2003). Extensive repetitive DNA facilitates prokaryotic genome plasticity. *Proc. Natl. Acad. Sci. U.S.A.* 100 13579–13584. 10.1073/pnas.1735481100 14593200 PMC263856

[B4] Bäckdahl J. Franzén L. Massier L. Li Q. Jalkanen J. Gao H. (2021). Spatial mapping reveals human adipocyte subpopulations with distinct sensitivities to insulin. *Cell Metab.* 33:1869–1882.e6. 10.1016/j.cmet.2021.07.018 34380013

[B5] Barone M. Garelli S. Rampelli S. Agostini A. Matysik S. D’Amico F. (2022). Multi-omics gut microbiome signatures in obese women: Role of diet and uncontrolled eating behavior. *BMC Med.* 20:500. 10.1186/s12916-022-02689-3 36575453 PMC9795652

[B6] Cani P. Van Hul M. Lefort C. Depommier C. Rastelli M. Everard A. (2019). Microbial regulation of organismal energy homeostasis. *Nat. Metab.* 1 34–46. 10.1038/s42255-018-0017-4 32694818

[B7] Chambers E. Viardot A. Psichas A. Morrison D. Murphy K. Zac-Varghese S. (2015). Effects of targeted delivery of propionate to the human colon on appetite regulation, body weight maintenance and adiposity in overweight adults. *Gut* 64 1744–1754. 10.1136/gutjnl-2014-307913 25500202 PMC4680171

[B8] Chen Y. Shen X. Ma T. Yu X. Kwok L. Li Y. (2023). Adjunctive probio-X treatment enhances the therapeutic effect of a conventional drug in managing type 2 diabetes mellitus by promoting short-chain fatty acid-producing bacteria and bile acid pathways. *mSystems* 8:e0130022. 10.1128/msystems.01300-22 36688679 PMC9948714

[B9] Cong J. Zhou P. Zhang R. (2022). Intestinal microbiota-derived short chain fatty acids in host health and disease. *Nutrients* 14:1977. 10.3390/nu14091977 35565943 PMC9105144

[B10] de Vos W. Tilg H. Van Hul M. Cani P. (2022). Gut microbiome and health: Mechanistic insights. *Gut* 71 1020–1032. 10.1136/gutjnl-2021-326789 35105664 PMC8995832

[B11] Depner M. Taft D. Kirjavainen P. Kalanetra K. Karvonen A. Peschel S. (2020). Maturation of the gut microbiome during the first year of life contributes to the protective farm effect on childhood asthma. *Nat. Med.* 26 1766–1775. 10.1038/s41591-020-1095-x 33139948

[B12] Fackelmann G. Manghi P. Carlino N. Heidrich V. Piccinno G. Ricci L. (2025). Gut microbiome signatures of vegan, vegetarian and omnivore diets and associated health outcomes across 21,561 individuals. *Nat. Microbiol.* 10 41–52. 10.1038/s41564-024-01870-z 39762435 PMC11726441

[B13] Fan Y. Pedersen O. (2021). Gut microbiota in human metabolic health and disease. *Nat. Rev. Microbiol.* 19 55–71. 10.1038/s41579-020-0433-9 32887946

[B14] Guzior D. Okros M. Shivel M. Armwald B. Bridges C. Fu Y. (2024). Bile salt hydrolase acyltransferase activity expands bile acid diversity. *Nature* 626 852–858. 10.1038/s41586-024-07017-8 38326608

[B15] Hall B. Levy S. Dufault-Thompson K. Arp G. Zhong A. Ndjite G. (2024). BilR is a gut microbial enzyme that reduces bilirubin to urobilinogen. *Nat. Microbiol.* 9 173–184. 10.1038/s41564-023-01549-x 38172624 PMC10769871

[B16] He B. Xiong Y. Hu T. Zong M. Wu H. (2023). *Bifidobacterium* spp. as functional foods: A review of current status, challenges, and strategies. *Crit. Rev. Food Sci. Nutr.* 63 8048–8065. 10.1080/10408398.2022.2054934 35319324

[B17] Hill J. Bell R. Barrios L. Baird H. Ost K. Greenewood M. (2025). Neonatal fungi promote lifelong metabolic health through macrophage-dependent β cell development. *Science* 387:eadn0953. 10.1126/science.adn0953 40048508 PMC12036834

[B18] Hong J. Jia Y. Pan S. Jia L. Li H. Han Z. (2016). Butyrate alleviates high fat diet-induced obesity through activation of adiponectin-mediated pathway and stimulation of mitochondrial function in the skeletal muscle of mice. *Oncotarget* 7 56071–56082. 10.18632/oncotarget.11267 27528227 PMC5302897

[B19] Hou K. Wu Z. Chen X. Wang J. Zhang D. Xiao C. (2022). Microbiota in health and diseases. *Signal. Transduct. Target Ther.* 7:135. 10.1038/s41392-022-00974-4 35461318 PMC9034083

[B20] International Diabetes Federation (2025). *IDF diabetes atlas 2025.* Available online at: https://diabetesatlas.org/resources/idf-diabetes-atlas-2025/ (accessed May 28, 2025).

[B21] Jia W. Chan J. Wong T. Fisher E. (2025). Diabetes in China: Epidemiology, pathophysiology and multi-omics. *Nat. Metab.* 7 16–34. 10.1038/s42255-024-01190-w 39809974

[B22] Jia W. Li Y. Cheung K. Zheng X. (2024). Bile acid signaling in the regulation of whole body metabolic and immunological homeostasis. *Sci. China Life Sci.* 67 865–878. 10.1007/s11427-023-2353-0 37515688

[B23] Jin J. He Y. Guo J. Pan Q. Wei X. Xu C. (2023). BACH1 controls hepatic insulin signaling and glucose homeostasis in mice. *Nat. Commun.* 14:8428. 10.1038/s41467-023-44088-z 38129407 PMC10739811

[B24] Karlsson F. Tremaroli V. Nookaew I. Bergström G. Behre C. Fagerberg B. (2013). Gut metagenome in European women with normal, impaired and diabetic glucose control. *Nature* 498 99–103. 10.1038/nature12198 23719380

[B25] Kimura I. Ozawa K. Inoue D. Imamura T. Kimura K. Maeda T. (2013). The gut microbiota suppresses insulin-mediated fat accumulation via the short-chain fatty acid receptor GPR43. *Nat. Commun.* 4:1829. 10.1038/ncomms2852 23652017 PMC3674247

[B26] Kootte R. Levin E. Salojärvi J. Smits L. Hartstra A. Udayappan S. (2017). Improvement of insulin sensitivity after lean donor feces in metabolic syndrome is driven by baseline intestinal microbiota composition. *Cell Metab.* 26:611–619.e6. 10.1016/j.cmet.2017.09.008 28978426

[B27] Lapébie P. Lombard V. Drula E. Terrapon N. Henrissat B. (2019). Bacteroidetes use thousands of enzyme combinations to break down glycans. *Nat. Commun.* 10:2043. 10.1038/s41467-019-10068-5 31053724 PMC6499787

[B28] Layden B. Angueira A. Brodsky M. Durai V. Lowe W. (2013). Short chain fatty acids and their receptors: New metabolic targets. *Transl. Res.* 161 131–140. 10.1016/j.trsl.2012.10.007 23146568

[B29] Li H. Zhang L. Li J. Wu Q. Qian L. He J. (2024). Resistant starch intake facilitates weight loss in humans by reshaping the gut microbiota. *Nat. Metab.* 6 578–597. 10.1038/s42255-024-00988-y 38409604 PMC10963277

[B30] Li M. Wang Y. Guo C. Wang S. Zheng L. Bu Y. (2023). The claim of primacy of human gut *Bacteroides ovatus* in dietary cellobiose degradation. *Gut Microbes* 15:2227434. 10.1080/19490976.2023.2227434 37349961 PMC10291918

[B31] Liu C. Du M. Xie L. Wang W. Chen B. Yun C. (2024). Gut commensal *Christensenella minuta* modulates host metabolism via acylated secondary bile acids. *Nat. Microbiol.* 9 434–450. 10.1038/s41564-023-01570-0 38233647

[B32] Liu R. Hong J. Xu X. Feng Q. Zhang D. Gu Y. (2017). Gut microbiome and serum metabolome alterations in obesity and after weight-loss intervention. *Nat. Med.* 23 859–868. 10.1038/nm.4358 28628112

[B33] Lloyd L. (2024). Mellow yellow: BilR reduces bilirubin to urobilinogen. *Nat. Rev. Urol.* 21:125. 10.1038/s41585-024-00863-1 38355923

[B34] Lopera-Maya E. Kurilshikov A. van der Graaf A. Hu S. Andreu-Sánchez S. Chen L. (2022). Effect of host genetics on the gut microbiome in 7,738 participants of the Dutch microbiome project. *Nat. Genet.* 54 143–151. 10.1038/s41588-021-00992-y 35115690

[B35] Mocanu V. Zhang Z. Deehan E. Kao D. Hotte N. Karmali S. (2021). Fecal microbial transplantation and fiber supplementation in patients with severe obesity and metabolic syndrome: A randomized double-blind, placebo-controlled phase 2 trial. *Nat. Med.* 27 1272–1279. 10.1038/s41591-021-01399-2 34226737

[B36] Mohanty I. Mannochio-Russo H. Schweer J. El Abiead Y. Bittremieux W. Xing S. (2024). The underappreciated diversity of bile acid modifications. *Cell* 187:1801–1818.e20. 10.1016/j.cell.2024.02.019 38471500 PMC12248420

[B37] Moran B. Flatt P. McKillop A. M. (2016). G protein-coupled receptors: Signalling and regulation by lipid agonists for improved glucose homoeostasis. *Acta Diabetol.* 53 177–188. 10.1007/s00592-015-0826-9 26739335

[B38] Muzurović E. Mikhailidis D. Mantzoros C. (2021). Non-alcoholic fatty liver disease, insulin resistance, metabolic syndrome and their association with vascular risk. *Metabolism* 119:154770. 10.1016/j.metabol.2021.154770 33864798

[B39] Nie Q. Sun Y. Li M. Zuo S. Chen C. Lin Q. (2023). Targeted modification of gut microbiota and related metabolites via dietary fiber. *Carbohydr. Polym.* 316:120986. 10.1016/j.carbpol.2023.120986 37321707

[B40] Nshanian M. Gruber J. Geller B. Chleilat F. Lancaster S. White S. (2025). Short-chain fatty acid metabolites propionate and butyrate are unique epigenetic regulatory elements linking diet, metabolism and gene expression. *Nat. Metab.* 7 196–211. 10.1038/s42255-024-01191-9 39789354 PMC11774759

[B41] Peng L. He Z. Chen W. Holzman I. Lin J. (2007). Effects of butyrate on intestinal barrier function in a Caco-2 cell monolayer model of intestinal barrier. *Pediatr. Res.* 61 37–41. 10.1203/01.pdr.0000250014.92242.f3 17211138

[B42] Qin Y. Havulinna A. Liu Y. Jousilahti P. Ritchie S. Tokolyi A. (2022). Combined effects of host genetics and diet on human gut microbiota and incident disease in a single population cohort. *Nat. Genet.* 54 134–142. 10.1038/s41588-021-00991-z 35115689 PMC9883041

[B43] Qu Q. He P. Zhang Y. Yang S. Zeng P. (2024). The intervention of probiotics on type 2 diabetes mellitus in animal models. *Mol. Nutr. Food Res.* 68:e2200815. 10.1002/mnfr.202200815 37967330

[B44] Ridaura V. Faith J. Rey F. Cheng J. Duncan A. Kau A. (2013). Gut microbiota from twins discordant for obesity modulate metabolism in mice. *Science* 341:1241214. 10.1126/science.1241214 24009397 PMC3829625

[B45] Rollenske T. Burkhalter S. Muerner L. von Gunten S. Lukasiewicz J. Wardemann H. (2021). Parallelism of intestinal secretory IgA shapes functional microbial fitness. *Nature* 598 657–661. 10.1038/s41586-021-03973-7 34646015

[B46] Sanna S. van Zuydam N. Mahajan A. Kurilshikov A. Vich Vila A. Võsa U. (2019). Causal relationships among the gut microbiome, short-chain fatty acids and metabolic diseases. *Nat. Genet.* 51 600–605. 10.1038/s41588-019-0350-x 30778224 PMC6441384

[B47] Sato Y. Atarashi K. Plichta D. Arai Y. Sasajima S. Kearney S. (2021). Novel bile acid biosynthetic pathways are enriched in the microbiome of centenarians. *Nature* 599 458–464. 10.1038/s41586-021-03832-5 34325466

[B48] Schott C. Germain A. Lacombe J. Pata M. Faubert D. Boulais J. (2024). GAS6 and AXL promote insulin resistance by rewiring insulin signaling and increasing insulin receptor trafficking to endosomes. *Diabetes* 73 1648–1661. 10.2337/db23-0802 39046834

[B49] Schwiertz A. Taras D. Schäfer K. Beijer S. Bos N. Donus C. (2010). Microbiota and SCFA in lean and overweight healthy subjects. *Obesity (Silver Spring)* 18 190–195. 10.1038/oby.2009.167 19498350

[B50] Sonnert N. Rosen C. Ghazi A. Franzosa E. Duncan-Lowey B. González-Hernández J. (2024). A host-microbiota interactome reveals extensive transkingdom connectivity. *Nature* 628 171–179. 10.1038/s41586-024-07162-0 38509360

[B51] Sorbara M. Pamer E. (2022). Microbiome-based therapeutics. *Nat. Rev. Microbiol.* 20 365–380. 10.1038/s41579-021-00667-9 34992261

[B52] Takeuchi T. Kubota T. Nakanishi Y. Tsugawa H. Suda W. Kwon A. (2023). Gut microbial carbohydrate metabolism contributes to insulin resistance. *Nature* 621 389–395. 10.1038/s41586-023-06466-x 37648852 PMC10499599

[B53] Wang D. Doestzada M. Chen L. Andreu-Sánchez S. van den Munckhof I. Augustijn H. (2021). Characterization of gut microbial structural variations as determinants of human bile acid metabolism. *Cell Host Microbe* 29:1802–1814.e5. 10.1016/j.chom.2021.11.003 34847370

[B54] Wang H. Wang P. Wang X. Wan Y. Liu Y. (2012). Butyrate enhances intestinal epithelial barrier function via up-regulation of tight junction protein Claudin-1 transcription. *Dig. Dis. Sci.* 57 3126–3135. 10.1007/s10620-012-2259-4 22684624

[B55] Wang S. Ren H. Zhong H. Zhao X. Li C. Ma J. (2022). Combined berberine and probiotic treatment as an effective regimen for improving postprandial hyperlipidemia in type 2 diabetes patients: A double blinded placebo controlled randomized study. *Gut Microbes* 14:2003176. 10.1080/19490976.2021.2003176 34923903 PMC8726654

[B56] Wang T. Lu J. Shi L. Chen G. Xu M. Xu Y. (2020). Association of insulin resistance and β-cell dysfunction with incident diabetes among adults in China: A nationwide, population-based, prospective cohort study. *Lancet Diabetes Endocrinol.* 8 115–124. 10.1016/S2213-8587(19)30425-5 31879247

[B57] Wardman J. Bains R. Rahfeld P. Withers S. (2022). Carbohydrate-active enzymes (CAZymes) in the gut microbiome. *Nat. Rev. Microbiol.* 20 542–556. 10.1038/s41579-022-00712-1 35347288

[B58] Wu G. Xu T. Zhao N. Lam Y. Ding X. Wei D. (2024). A core microbiome signature as an indicator of health. *Cell* 187:6550–6565.e11. 10.1016/j.cell.2024.09.019 39378879

[B59] Yadav H. Lee J. Lloyd J. Walter P. Rane S. (2013). Beneficial metabolic effects of a probiotic via butyrate-induced GLP-1 hormone secretion. *J. Biol. Chem.* 288 25088–25097. 10.1074/jbc.M113.452516 23836895 PMC3757173

[B60] Yan X. Li J. Wu D. (2023). The role of short-chain fatty acids in acute pancreatitis. *Molecules* 28:4985. 10.3390/molecules28134985 37446647 PMC10343743

[B61] Ye M. Yu J. Shi X. Zhu J. Gao X. Liu W. (2021). Polysaccharides catabolism by the human gut bacterium – *Bacteroides thetaiotaomicron*: Advances and perspectives. *Crit. Rev. Food Sci. Nutr.* 61 3569–3588. 10.1080/10408398.2020.1803198 32779480

[B62] Yin Q. da Silva A. Zorrilla F. Almeida A. Patil K. Almeida A. (2025). Ecological dynamics of *Enterobacteriaceae* in the human gut microbiome across global populations. *Nat. Microbiol.* 10 541–553. 10.1038/s41564-024-01912-6 39794474 PMC11790488

[B63] Zaky A. Glastras S. Wong M. Pollock C. Saad S. (2021). the role of the gut microbiome in diabetes and obesity-related kidney disease. *Int. J. Mol. Sci.* 22:9641. 10.3390/ijms22179641 34502562 PMC8431784

[B64] Zhang H. Cao H. Wang Y. Xin F. (2020). Research progress on carbohydrate active enzymes (CAZYmes) derived from human gut microbiota. *Prog. Biochem. Biophys.* 47 1–19.

[B65] Zhang X. Chen J. Yi K. Peng L. Xie J. Gou X. (2020). Phlorizin ameliorates obesity-associated endotoxemia and insulin resistance in high-fat diet-fed mice by targeting the gut microbiota and intestinal barrier integrity. *Gut Microbes* 12 1–18. 10.1080/19490976.2020.1842990 33222603 PMC7714487

[B66] Zhang Q. Schwarz D. Cheng Y. Sohrabi Y. (2024). Unraveling host genetics and microbiome genome crosstalk: A novel therapeutic approach. *Trends Mol. Med.* 30 1007–1009. 10.1016/j.molmed.2024.06.007 38937208

[B67] Zhang Y. Luo K. Peters B. Mossavar-Rahmani Y. Moon J. Wang Y. (2025). Sugar-sweetened beverage intake, gut microbiota, circulating metabolites, and diabetes risk in hispanic community health study/study of latinos. *Cell Metab.* 37:578–591.e4. 10.1016/j.cmet.2024.12.004 39892390 PMC11885037

[B68] Zhao L. Zhang F. Ding X. Wu G. Lam Y. Wang X. (2018). Gut bacteria selectively promoted by dietary fibers alleviate type 2 diabetes. *Science* 359 1151–1156. 10.1126/science.aao5774 29590046

[B69] Zhernakova D. Wang D. Liu L. Andreu-Sánchez S. Zhang Y. Ruiz-Moreno A. (2024). Host genetic regulation of human gut microbial structural variation. *Nature* 625 813–821. 10.1038/s41586-023-06893-w 38172637 PMC10808065

[B70] Zhou Q. Sun W. Chen J. Zhang H. Liu J. Lin Y. (2022). Phenylalanine impairs insulin signaling and inhibits glucose uptake through modification of IRβ. *Nat. Commun.* 13:4291. 10.1038/s41467-022-32000-0 35879296 PMC9314339

