# Supplementary material for: The role of gut microbiota in insulin resistance: recent progress
Source: Front Microbiol. 2025 Jul 25;16:1633029. doi: 10.3389/fmicb.2025.1633029 (PMC12332526; doi:10.3389/fmicb.2025.1633029)
Supplement: Supplementary file 1 [file Table_1.docx]

| **Bacteria and Genera** | **Metabolite** | **Representative Substances** | **Physiological Functions** |
| --- | --- | --- | --- |
| *Bacteroides,Firmicutes,Akkermansia muciniphila* | SCFAs | Acetate,Propionate,Butyrate | Maintain intestinal barrier integrity, exert anti-inflammatory effects, regulate immune function, provide energy for intestinal epithelium, enhance insulin sensitivity(16, 17) |
| *Clostridium,Bacteroides,Christensenella minuta* | secondarybile acids | Deoxycholic Acid,Lithocholic Acid | Regulate lipid metabolism, modulate host bile acid signaling pathway(51-54) |
|  |  | 3-O-acylated secondary bile acids | Targetedly inhibit intestinal FXR, regulate the gut-liver signaling axis, alleviate glucose and lipid metabolic disorders(49) |
| *Lactic acid bacteria, Bifidobacterium,Clostridium* | tryptophan metabolites | Indole,Indole-3-propionic acid,5-Hydroxytryptophan | Modulate immune responses, maintain intestinal barrier integrity, facilitate neurotransmitter synthesis (e.g., serotonin) |
| *Bacteroides* | histidine derivatives | imidazole propionate | Inhibit insulin receptor signaling(46) |
| *Bacteroides,Escherichiacoli* | vitamin | Vitamin K,B vitamins (e.g., B12) | Mediate coagulation processes, regulate energy metabolism, support neurological function |
| *Escherichia coli ,Lactobacillus* | phenylalanine | phenylalanine | positively correlated with HOMA-IR index(47) |
